# Inflammation as a Keystone of Bone Marrow Stroma Alterations in Primary Myelofibrosis

**DOI:** 10.1155/2015/415024

**Published:** 2015-11-12

**Authors:** Christophe Desterke, Christophe Martinaud, Nadira Ruzehaji, Marie-Caroline Le Bousse-Kerdilès

**Affiliations:** ^1^Inserm UMS33, Paul Brousse Hospital, 14 Avenue Paul-Vaillant Couturier, 94800 Villejuif, France; ^2^Department of Clinical Biology, HIA Percy, 101 Avenue Henri Barbusse, 92140 Clamart, France; ^3^Inserm UMR-S1197, Paul Brousse Hospital, 14 Avenue Paul-Vaillant Couturier, 94800 Villejuif, France; ^4^French Intergroup on Myeloproliferative Neoplasms (FIM), France; ^5^GDR 2697 Micronit, France

## Abstract

Primary myelofibrosis (PMF) is a clonal myeloproliferative neoplasm where severity as well as treatment complexity is mainly attributed to a long lasting disease and presence of bone marrow stroma alterations as evidenced by myelofibrosis, neoangiogenesis, and osteosclerosis. While recent understanding of mutations role in hematopoietic cells provides an explanation for pathological myeloproliferation, functional involvement of stromal cells in the disease pathogenesis remains poorly understood. The current dogma is that stromal changes are secondary to the cytokine “storm” produced by the hematopoietic clone cells. However, despite therapies targeting the myeloproliferation-sustaining clones, PMF is still regarded as an incurable disease except for patients, who are successful recipients of allogeneic stem cell transplantation. Although the clinical benefits of these inhibitors have been correlated with a marked reduction in serum proinflammatory cytokines produced by the hematopoietic clones, further demonstrating the importance of inflammation in the pathological process, these treatments do not address the role of the altered bone marrow stroma in the pathological process. In this review, we propose hypotheses suggesting that the stroma is inflammatory-imprinted by clonal hematopoietic cells up to a point where it becomes “independent” of hematopoietic cell stimulation, resulting in an inflammatory vicious circle requiring combined stroma targeted therapies.

## 1. Introduction

Hematopoiesis is orchestrated through a tightly regulated network of events including cell-cell interactions, cytokines, chemokines, proteases, and extracellular matrix components within an environment where oxygen level and calcium concentration are monitored. At steady state, adult HSCs reside in the BM in specialized niches made up of bone and vascular and nervous structures [1, 2]. Within these niches, the balance between HSC quiescence, self-renewal, and differentiation is controlled by a sophisticated dialogue between HSCs, stromal and neural cells in a “seed (stem cells) and soil (stroma)” relationship. This equilibrium must be tightly controlled since its disruption can participate in the emergence/development of hematological malignancies such as myelodysplastic and myeloproliferative disorders [3–5].

Primary myelofibrosis (PMF) is a clonal myeloproliferative neoplasm (MPN) of the elderly whose severity as well as treatment complexity is mainly attributed to the fact that PMF is a long lasting disease and to the presence of profound changes in the bone marrow (BM) stroma evidenced by myelofibrosis, neoangiogenesis, and osteosclerosis [6]. Despite new therapies targeting the myeloproliferation, PMF is still regarded as an incurable disease except for patients who are successful recipients of allogeneic stem cell transplantation.

This may, in part, be due to the fact that current therapies are unable to influence the altered stroma and to reestablish efficient hematopoiesis requiring the elimination of neoplastic hematopoietic cells. Actually, with the exception of ruxolitinib in case reports, most JAK2 inhibitors, despite being effective in alleviating constitutional symptoms, have no or very few effects on bone marrow fibrosis [7]. Whereas there is no study analyzing the direct effect of JAK2 inhibitors on stromal cells, these inhibitors have been mainly designed to suppress the cytokine signalling cascade caused by the constitutive activation of JAK2. However, by providing significant improvements in splenomegaly, associated clinical manifestations, and disease related constitutional symptoms, their clinical benefits have been associated with a marked reduction in serum proinflammatory cytokines produced in particular by the hematopoietic cells, demonstrating the importance of inflammation in the pathological process [8]. More recently, preclinical studies have observed that ruxolitinib causes a rapid and prolonged decrement of T regulatory cells and impairs the normal function of dendritic cells suggesting that JAK2 inhibitors can also act via an immunosuppressive effect [9–11].

The development of novel more effective therapies will also depend on a better understanding of the disease pathogenesis. Although current knowledge about the role of mutations in hematopoietic cells partially explains myeloproliferation, functional involvement of stromal cells in PMF pathogenesis remains poorly understood. Up to date, the dogma is that stromal changes, including myelofibrosis that is the hallmark of the disease, are secondary to the cytokine “storm” created by hematopoietic cells from the clone and especially by pathological megakaryocytes (MKs) [14]. This assumption is mainly based on the lack of information on molecular anomalies in stromal cells and does not take into account the possibility for stromal cells to acquire functional abnormalities within the inflammatory process that is developed during the course of the disease. Actually, an increasing number of results from our laboratory suggest the role of an altered dialogue between hematopoietic and stromal cells in the pathogenesis of PMF at the origin of our “bad seeds in bad soil” concept [6, 15–18]. Hence, during the long lasting process of PMF, hematopoietic, immune, and mesenchymal stromal cells could be both effective and responsive cells, creating a vicious circle that is difficult to break by current therapies.

Understanding the mechanisms by which the “bad soil” (stromal cells) contributes and responds to the inflammatory process participating in making the bed for the “bad seeds” (clonal hematopoietic cells) would therefore help in the development of new immune- and cell-based therapies. By targeting inflammation and restoring stroma homeostasis, these new treatments will synergize with the current drugs mainly focused on eradicating the malignant hematopoietic clones.

In this review, based on hypotheses from our group, we will consider arguments concerning the role of inflammation as a driving mechanism for “intrinsic” (i.e., HSC-independent) alterations of mesenchymal stromal cells in PMF patients. We will bring some controversies on the pathogenesis of this no longer “forgotten myeloproliferative disorder” [19], but still misunderstood neoplasm.

## 2. Myeloproliferation and Myelofibrosis: The Dual Complementarity of Primary Myelofibrosis?

According to the 2008 WHO classification, primary myelofibrosis belongs to Philadelphia negative myeloproliferative neoplasms [20]. Together with Polycythemia Vera (PV) and Essential Thrombocythemia (ET), PMF shares features of myeloproliferative diseases that is the expansion of clonal hematopoietic stem/progenitor cells. PMF is characterized by a shortened life expectancy, myelofibrosis, osteosclerosis, and extramedullary hematopoiesis [14, 21]. Diagnosis relies on clinical, biological, molecular, and bone marrow biopsy analysis. Clinical and biological data demonstrate splenomegaly, dacryocytosis, basophilia, or leukoerythroblastosis.

Several molecular mechanisms and other clues suggest the clonal nature of the disease and that mutational clonal evolution in PMF is dependent on multiple hematopoietic clones [22–24]. The pathological hematopoietic stem cells harbor genetic mutations conferring the proliferative phenotype of the disease. The* JAK2* V617F and* MPL* 515 mutations, present in about 50% and 5% of PMF cases, respectively, result in a permanent activation of the JAK/STAT signalling pathways, conferring* in vitro* altered sensitivity/independence of clones to growth factors [25–27]. The recently discovered Calreticulin mutations complete the scope of PMF mutations, occurring in 25% and 88% of patients without* MPL* and* JAK2* mutations [28]. Finally, less than 10% of patients are “triple-negative” [29]. It is suggested that, as* JAK2* and* MPL* mutations, the most frequent Calreticulin mutation (Exon 9 Calreticulin type 1 mutation) confers a relative independence of the clonal cells to growth factors [30]. Calreticulin protein is involved in intracytoplasmic protein trafficking and mutations could alter membrane expression of receptors participating in the proliferative abilities of clonal cells [31, 32]. Other mutations can occur less frequently and participate in the activation of the JAK/STAT pathways: for instance, LNK, an adaptor protein which negatively regulates TPO signalling, is mutated in some patients [33] or promoters of tumor-suppressor genes like SOCS-3 which are hypermethylated [34]. Apart from the abovementioned mutations, others such as* NRAS* and* NF1* mutations in the MAP-kinase pathways are associated with worse prognosis [35, 36]. Mutations can also occur in epigenetic regulator genes such as* TET-2* [37],* DNMT3A* [38], or* ASXL1* [39]. Recently, stem cell populations from PMF patients identified by the expression of CD133 have been investigated and after transplantation into mice were able to recapitulate major PMF parameters, revealing that CD133 marks a stem cell population that drives PMF [24]. However, despite numerous mutations, none are able, as the* BCR-ABL* mutations in chronic myeloid leukemia, to fully recapitulate the disease in an animal model or to entirely explain the pathophysiological features of PMF.

To decipher the natural course of the disease, clonal cells must be replaced in their environment and time scale should be considered. The concept that hematopoietic stem cells are intimately dependent on interactions with their environment has emerged in the late 70s [40] and became preeminent in the last few years [41]. Actually, as described in the introduction, HSC cell fate is highly dependent on cell-to-cell connections, matrix-to-cell interactions, and chemokine stimulation. Those cellular and noncellular elements are key components of the so-called “hematopoietic niches” [42]. Three “distinct” niches are conceptually identified. The first one is the endosteal niche, which is located close to the endosteum and whose main component is the Shaped N-Cadherin positive osteoblast [43] and where HSC quiescence is maintained [44]. In contrast, the vascular niche and the CXCL-12 abundant perivascular cells [45] would be the place of differentiation and proliferation [46]. A third niche would be the link between those specialized areas, integrating signals from nervous system through Schwann cells [47]. The mesenchymal stromal cells (MSCs) would be the prominent components of this niche [48]. Through their ability to differentiate into fibroblasts, osteoblasts, and adipocytes and to produce extracellular matrix elements, MSCs are reported be milestone regulators of hematopoiesis, questioning their potential role in hematopoietic malignancies.

In recent years, abnormalities in the BM microenvironment have appeared as critical promoters of myeloid malignancies. In murine models, genetic ablation of the retinoic acid receptor gamma (*Rar-γ*) or retinoblastoma (*Rb*) genes in BM stromal cells have been reported to promote MPN development [49, 50], whereas inactivation of the microRNA-processing enzyme dicer in immature OSTERIX- (OSX) expressing osteoprogenitors caused myelodysplastic syndrome (MDS) [4]. Interestingly, Wei et al. have shown that the murine microenvironment determines the lineage outcome of the human biphenotypic MLL-AF9 leukemia stem cells when graphed in immunodeficient mice [51]. In humans, evidences are scantier. One of the most intriguing piece of data is the development of donor cell leukemia in recipients of hematopoietic stem cell transplantations, with the same phenotype of the former disease, strongly suggesting the role of recipient microenvironment in the onset of the disease [52]. Analysis of beta-catenin expression in osteoblasts of patients with myelodysplastic syndrome or myeloid leukemia also revealed that the microenvironment might interact with hematopoietic cells in the development of the disease [5].

**Figure 1:**
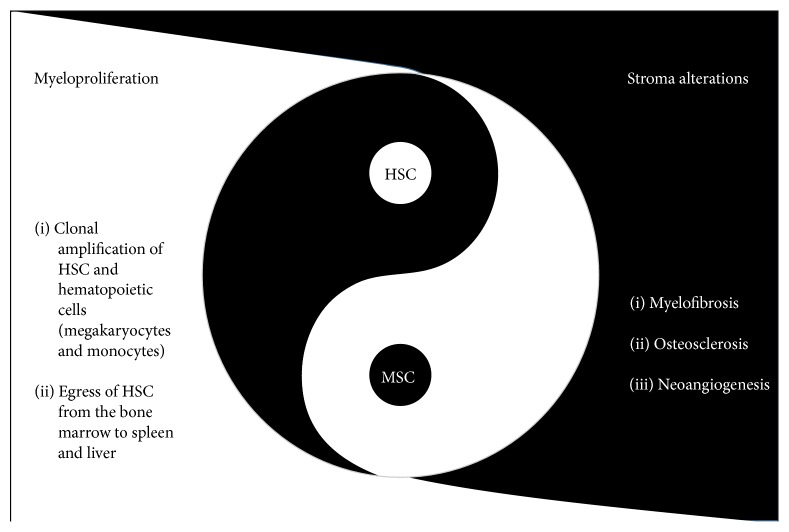
Primary myelofibrosis: the dual complementarity of hematopoietic and stromal stem cells. PMF is characterized by medullar and extramedullary clonal expansion of hematopoietic stem cells (HSCs) and dystrophic megakaryocytes (MKs), altogether with myelofibrosis and osteosclerosis involving fibroblasts and osteoblasts, as well as neoangiogenesis. These elements stand together by growth factors and inflammatory cytokines mediated interactions [6].

In PMF, several evidences argue for an impaired microenvironment in relation with inflammation. As previously mentioned examination of BM biopsies represents a key step in the PMF diagnosis. Besides the myeloproliferation, especially megakaryocytic proliferation with abnormal morphological features, PMF is characterized by myelofibrosis, neoangiogenesis, and osteosclerosis. Megakaryocytes [12] and monocytes [53] derived from the malignant clones produce high levels of Transforming Growth Factor-beta1 (TGF-*β*1) [54], Platelet-Derived Growth Factor (PDGF), basic Fibroblast Growth Factor (bFGF) [55], and Vascular Endothelial Growth Factor (VEGF) [56]. Particularly, TGF-*β*1 exerts profibrotic effects on fibroblasts and favors ossification by osteoblasts. Concomitantly, osteoprotegerin production by fibroblasts inhibits osteoclastogenesis and enhances bone marrow osteosclerosis. Neoangiogenesis associated with morphological modification of vessels and of pericytes is present in the bone marrow of PMF patients [57]. Endothelial cells of spleen vessels harbor* JAK2* mutation [58] and are known to increase cellular adhesion [59], demonstrating that bone marrow modifications are not the sole elements of the microenvironment alterations in PMF. Actually, one of the features that distinguishes PMF from ET and PV is the extramedullary hematopoiesis in spleen and liver and high number of CD34^+^ circulating cells [60]. Disruption of the CXCL12-CXCR4 axis involved in this phenomenon is related to the abnormal methylation of the CXCR4 promoter [61] and with metalloproteinase deregulation in the bone marrow [62]. In the spleen of PMF patients, CD34^+^ cells are able to give rise to extramedullary hematopoiesis in a remodeled niche as evidenced by specific properties of fibroblasts isolated from patients [17, 18]. Altogether, these data demonstrate a wide disruption in the crosstalk between hematopoietic stem cells and their stromal microenvironment in PMF (Figure 1).

## 3. Inflammation: A Pathophysiologically Important Component of MPN Pathogenesis

Inflammation is a key pathophysiological component of a wide range of diseases [63], including PMF and the other Philadelphia-negative chronic MPNs [64]. Inflammation is a protective reaction in response to injury and its objective is to eliminate harmful stimulus or promote repair of damaged tissue, a phenomenon observed during wound healing [1]. It is important to distinguish between acute and chronic inflammation. The acute inflammatory response is a complex and coordinated sequence of events involving a large number of molecular and cellular changes. It begins with the production of soluble mediators including chemokines and cytokines secreted by resident cells and ends with the resolution or “switching off” of the inflammatory response leading to restoration of normal tissue homeostasis. Although the acute inflammatory response is critical for survival [63], dysregulation of this process may predispose certain individuals to the development of chronic inflammation. A prerequisite for inflammation resolution is to switch off or eradicate the primary stimulus that initiated it [63]. Failure to eradicate the initial trigger may lead to chronic inflammation as exemplified by MPNs, which is hypothesized to result from a sustained inflammation exacerbated by continuous release of proinflammatory cytokines and chemokines [64].

### 3.1. What Triggers Inflammation in MPNs?

It is believed that MPNs arise from mutant hematopoietic stem cells implying that these disorders are clonal hematologic diseases [2]. However, if MPNs are clonal stem cell diseases and* JAK2* mutation in the myeloproliferative disorders is not in the germ line but, rather, is acquired [2], then what is the nature of the primary trigger that causes the initial genetic defect? We know that inflammation in general occurs in response to something that destabilizes local homeostasis; in MPNs, identification of that “something” has been proven elusive. The precise nature of the initial trigger may remain unknown, but what remains certain is that the MPNs are associated with a chronic inflammatory state which is referred to as a “human inflammation model” with “inflamed bone marrow,” “inflamed stem cell niche,” and “inflamed circulation” [64].

### 3.2. Chronic Inflammation in PMF: What Can We Learn from Other Inflammatory Disorders?

Could a chronic inflammatory state that is triggered initially by a process other than infection, tissue injury, or autoimmunity be causing genomic instability and fibrosis in PMF? If the answer is yes, then it is tempting to compare PMF with atherosclerosis—class of diseases with nonresolving inflammation. PMF and atherosclerosis share two common characteristics. First and foremost, both atherosclerosis and PMF lack the potential for removing the inflammatory stimulus which would normally occur in most cases of infection or injury [65]. Secondly, both diseases are often associated with aging. Important advances in the treatment of atherosclerosis have been made [66]; hence in this context, what can we learn from the advances made in diseases in which inflammation is an important driving force? More importantly, how might the inflammatory nature of atherosclerosis lead to better understanding of pathological inflammation and new therapeutic opportunities in MPNs? The understanding of the pathology of nonresolving inflammation, which is typically initiated by pattern recognition receptors such as toll-like receptors (TLRs) that recognize pathogen-associated molecular patterns (PAMPs) and damage-associated molecular patterns (DAMPs) [67], leads to discovery of a class of anti-inflammatory drugs known as disease-modifying agents of rheumatoid diseases (DMARDs) [68], which are distinguished by their ability to reduce or prevent tissue damage caused by the inflammatory attack, especially when used early in the course of the disease. Just as in other inflammatory diseases including atherosclerosis [67], TLRs couple to signal transduction pathways that activate latent transcription factors that include members of the NF*κ*B and AP-families [65], which happen to be increased in hematopoietic cells and stroma cells, exposing these cells to a constant oxidative stress [64]. These factors in turn induce the expression of a large number of genes that aid chemokine release, which in turn regulate the recruitment of additional immune cells [64]. Increased TLR activity could result in augmented production of cytokines and chemokines activating leukocytes in the bone marrow to make TNF-*α* and IL-6. IL-6 is known to increase NF*κ*B and STAT3 causing inhibition of apoptosis and increased myeloproliferation, hence creating an environment favorable to malignant transformation and expansion [64, 69].

## 4. How Does TGF-*β* Contribute to Fibrosis in the Context of Inflammation?

TGF-*β*, the most critical regulator of pathological fibrosis, is overexpressed in all fibrotic tissues and it induces collagen production in cultured fibroblasts, regardless of their origin [70]. TGF-*β* is part of a superfamily of 33 members that includes BMPs, activins, inhibins, growth differentiation factors, and myostatin [71]. The three TGF-*β* isoforms are encoded by different genes; TGF-*β*1, TGF-*β*2, and TGF-*β*3, which are secreted as latent proteins, interact with the same receptor heterodimers, TGFR-1 (TGF-*β* receptor type-1, also known as ALK-5) and TGFR-2 (TGF-*β* receptor type-2) [70]. All three isoforms exert TGF-*β* signalization mainly via its canonical SMAD pathway, although TGF-*β* can also activate other pathways that are collectively referred to as noncanonical TGF-*β* pathways [72].

Bone marrow is a heterogeneous organ containing diverse cell types. In the BM of MPNs patients, TGF-*β* is believed to be produced by hematopoietic cells, including necrotic and viable megakaryocytes [15]—important source of latent TGF-*β* stored within the alpha-granules of these bone marrow cells [15]. An increasing number of niche components have now been identified revealing a complex network of cell and matrix interactions and signalling pathways, which together create a unique microenvironment with TGF-*β* being an integral part of this environment. Cell-cell and cell-matrix interactions with the BM are critical components of the orchestrated process of activation of latent TGF-*β*. Interaction between BM nestin^+^ MSCs and BM Schwann cells was identified as contributing to MPN pathogenesis [73]. Actually, nonmyelinating BM Schwann cells promote TGF-*β* activation by exposing the growth factor to proteolytic cleavage by metalloproteinases [73].

TGF-*β* production correlates with the progression of fibrotic diseases and TGF-*β* inhibition has been shown to reduce fibrotic processes in many experimental models [74]. TGF-*β* is unequivocally a prominent stimulus and regulator of extracellular matrix formation. It mediates fibroblast and endothelial cell proliferation, suggesting their involvement in the stromal reaction and reinforcing the hypothesis of a connection between fibrosis and angiogenesis as suggested in various fibrotic diseases including pulmonary and eye fibrosis as well as systemic sclerosis [15, 75]. TGF-*β* has been also implicated in the development of fibrosis associated with hematological disorders including hairy cell leukemia, acute megakaryoblastic leukemia, and PMF [15]. In PMF and other MPNs the stromal cells and fibroblasts responsible for the increased fibrosis, angiogenesis, and formation of new bone are not derived from the myeloproliferative clone [2]. BM microenvironment and its interactions with TGF-*β* have been proposed to contribute to myelofibrosis [76]. The question of how latent TGF-*β* becomes activated in the bone marrow of MPN patients is, therefore, central to the understanding and the treatment of fibrotic diseases. Although integrins [77] and thrombospondin-1 [78] have been known to activate latent TGF-*β* in other fibrotic disease models such as skin [70] and liver fibrosis [78], it is possible that this pattern of activation may also function in PMF (see Section 6).

Recently, based on transcriptomic analysis, Ciaffoni et al. have suggested that fibrosis in PMF may result from an autoimmune process triggered by dead megakaryocytes through activation on noncanonical TGF-*β* signaling [79]. The interesting assumption of autoimmunity as a possible cause of marrow fibrosis in PMF is reminiscent to historical articles such as those from Lang et al. [80] and Rondeau et al. [81] describing the presence of autoantibodies, their levels being related to the degree of fibrosis. However, whereas the parallel between apoptosis and fibrosis is of interest, the signification of the presence of autoantibodies in PMF patients as a “cause” or a consequence of the pathological mechanism is not clear. Since many recent studies suggest a positive association between autoimmune and inflammatory diseases and subsequent neoplasia development, this concern would merit extensive studies in an attempt to better combine immunomodulatory therapies to current treatments [82].

## 5. When Data-Mining Identifies MSCs as a Piece of the Inflammation Puzzle!

In PMF, the huge deregulation of inflammatory/fibrogenic cytokines is suggested to contribute to the clinical phenotype, including bone marrow fibrosis, increased angiogenesis, extramedullary hematopoiesis, constitutional symptoms, and cachexia. It has been suggested by Tefferi's group that plasma cytokine signature provides a useful laboratory tool for predicting and monitoring treatment response [83]. Interestingly, a two-cytokine (IL-8/sIL-2R*α*) based risk categorization stratified on a large cohort of patients has been shown to delineate different groups within specific DIPSS plus risk categories [83]. In patients, growth factors have been suggested to be mainly produced by dystrophic megakaryocytes and monocytes; however, recent data from our group also identified PMF MSCs, endothelial cells, and T lymphocytes as important sources of inflammatory cytokines [16, 84].

**Figure 2:**
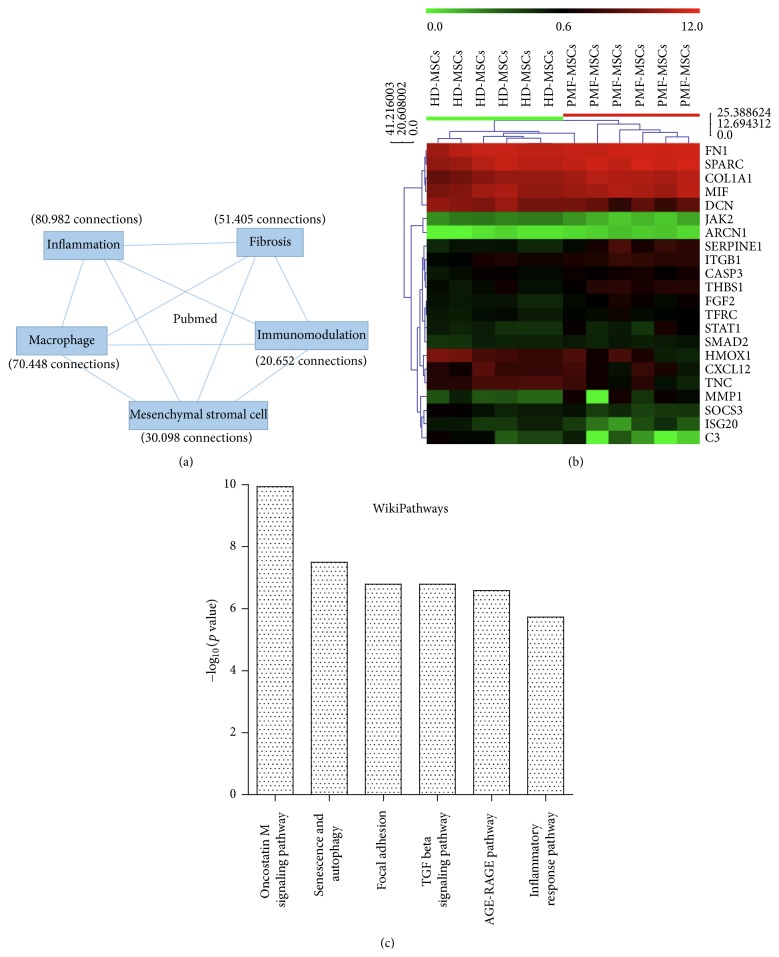
Data-mining prediction of inflammatory gene expression profile in BM-MSCs from PMF patients. (a) Keywords used during data-mining to link the scientific information between inflammation and altered niche in primary myelofibrosis; (b) unsupervised classification on data from inflammation prediction of gene expression profile from PMF BM-MSCs (transcriptome GSE44426); (c) functional enrichment on WikiPathway database for inflammation signature prediction of BM-MSCs from PMF patients.

To characterize inflammation in the altered bone marrow stroma from patients, we query information from the literature by data-mining using inflammation, fibrosis, macrophage, mesenchymal stromal cells, and immunomodulation as keywords (Figure 2). A total of 253.585 connections were collected between Pubmed and gene databases (Figure 2(a)). This collected information was crossed with the gene expression profile of BM-MSCs we performed in PMF patients (GSE44426) [85] in R software [86]. The inflammatory predictive signature allows performing an unsupervised classification and identified two distinct clusters of BM-MSC samples: PMF patients and healthy donors, demonstrating that BM-MSCs from PMF patients have a typical inflammatory gene expression profile which is different from their normal counterparts (Figure 2(b)). This data-mining analysis identified several altered pathways in PMF-MSCs that would be part of the pathophysiological process. Among them, inflammatory response, oncostatin M and TGF-*β* signalling pathways, focal adhesion, senescence, and autophagy are the most significant within the stromal niche context (Figure 2(c)).

**Figure 3:**
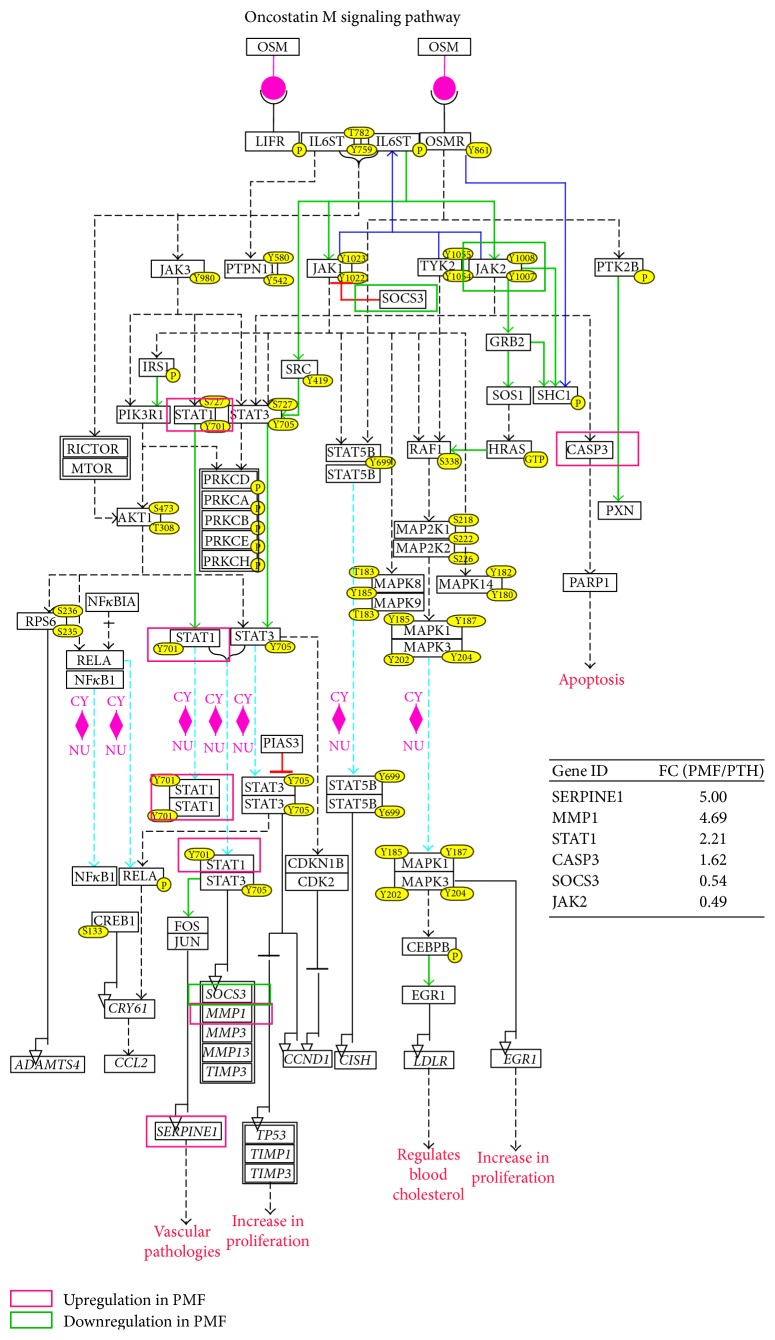
Oncostatin M signalization in mesenchymal stromal cells from PMF patients. WikiPathway diagram of oncostatin M signalization with drawing of genes deregulated in BM-MSCs from PMF patients (transcriptome GSE44426).

Oncostatin M (OSM), an interleukin-6-like inflammatory cytokine, is reported to play a role in a number of pathological processes including cancer. In MPNs such as PMF, activation of the JAK/STAT signalization resulting from the presence of the* JAK2 V617F* or* MPL 515* mutations in the hematopoietic lineage is known to stimulate OSM production by pathological megakaryocytes [87, 88]. In PMF-MSCs, the altered expression of genes such as* STAT1*,* SOCS3*,* MMP1,* and* SERPINE1* participating in the OSM signalling pathway suggests that they could be activated by OSM (Figure 3). The overexpression of* STAT1* (fold change = 2.21), an effector of signal transduction able to activate the expression of VEGF in response to OSM stimulation [89], evidences a possible link between BM-MSCs, OSM, and the increased VEGF expression [87] (Figure 3). Actually, a paracrine effect of oncostatin M could induce production of VEGF by the bone marrow stromal cells [87]. The massive neoangiogenesis [90] observed in association with the myelofibrosis in PMF patients is in agreement with such hypothesis. This is also confirmed in other Phi-negative myeloproliferative disorders such as PV and ET [91] where the plasma level of VEGF is correlated with the BM microvessel density [92].

*SERPINE1* is also highly upregulated (fold change = 5.0) in BM-MSCs from PMF patients. This molecule, also named Plasminogen Activator Inhibitor-1 (PAI-1), is known to be deregulated in PMF [93, 94] and to be associated with a bad prognosis in diverse cancers [95, 96]. Its role in vascular alterations [97], extracellular matrix reorganization [98], and metalloproteases regulation [99] through a TGF-*β*1 dependent mechanism [100] strengthens its potential participation to the stromal reaction and to the egress of hematopoietic progenitors from the BM observed in patients [62].

Inflammatory expression profile of PMF BM-MSCs highlights alterations of the senescence pathway regulation involving genes such as* SPARC*,* THBS1*,* FN1,* and* COL1A1*. In MPNs, expression of SPARC in BM stromal cells correlates with the degree of stromal changes and the severity of BM failure [101]. In a murine model of thrombopoietin-induced myelofibrosis using Sparc(−/−) mice and BM chimeras, SPARC contributes to the development of significant stromal fibrosis [101]. However, whereas in this thrombopoietin-induced myelofibrosis murine model, THBS1 is not required for TGF-*β*1 activation [102], it is suggested to be a mediator which discriminates PMF from ET patients within a profibrotic environment [103].

Together with thrombospondin and SPARC, tenascin forms a family of matrix proteins that caused a dose-dependent reduction in the number of focal adhesion-positive cells. Tenascin, observed in myelofibrosis with megakaryocytic hyperplasia, has a strong impact on chronic inflammation and on TGF-*β* activation and signalling [104]. This is in line with the notion that tenascin synthesis in BM fibroblasts is stimulated by TGF-*β* also produced by MK cells [105]. Connections between focal adhesions and proteins of the EMC involve integrins. Actually, integrin *β*1 participates in (1) mediating activation of latent TGF-*β* via ECM contraction and (2) modulating collagen production via a focal adhesion kinase/rac1/nicotinamide adenine dinucleotide phosphate oxidase (NOX)/reactive oxygen species (ROS) pathway. Therefore, multiple alterations of ECM and focal adhesion components like integrins observed in the BM could participate in activation of the TGF-*β* signalization in PMF patients.

Altogether results from this data-mining analysis suggest that chronic inflammation present in BM environment of PMF patients could induce a hypersensibility of MSCs to inflammatory molecules participating in creating a vicious circle. Additionally to TGF-*β* signals, BM-MSC hyperresponsiveness resulting from inflammation could result in liberation/activation of effectors contributing to (i) fibrosis (collagens, fibronectin, and tenascin C), (ii) extracellular matrix modeling (SERPINE1, MMP1), (iii) angiogenesis (oncostatin M signalling pathway), and (iv) hematopoietic progenitor homing/egress (CXCL12). Interestingly, as a demonstration of the role of “inflamm-aging” in BM stromal alterations, MSCs from patients also harbored changes linked to aging such as senescence, hypoxia, and AGEs/RAGE signalling pathways (Figure 3).

## 6. Inflammation as a Keystone of Bone Marrow Stroma Alterations in PMF

In PMF, bone marrow stroma alterations occur at cellular and noncellular level. Inflammation impacts cellular components of the hematopoietic niche: fibroblasts, osteoblasts, endothelial cells, and MSCs. Basic FGF is able to induce MSC proliferation and to act as an angiogenic growth factor [55, 106]. Interleukine-1 can also modulate fibroblastic abilities [107]. PDGF induces proliferation of fibroblastic cells [108], major producers of matrix components. In association with TGF-*β*1, this results in an increase of proteoglycans, fibronectin, and collagens. TGF-*β*1 is a powerful inducer of matrix-associated genes expression [109]. Concomitantly, it inhibits matrix proteases, leading to deep changes in the extracellular matrix (ECM) properties [110]. ECM remodeling could participate in alterations of hematopoiesis: megakaryopoiesis is stimulated by glycosaminoglycans [111], and some heparan sulfate proteoglycans are involved in myeloproliferation [112]. TGF-*β*1 is a potent inducer of GAG expression by osteoblasts [110], and on the other hand, GAGs could interact with Bone Morphogenic Proteins (BMPs) and induce osteogenic differentiation of MSCs [113]. Remodeling of ECM is of major importance, since matrix to cell interactions can modulate cell fate. For instance, modification of physical traction forces in the ECM can participate in the shift of TGF-*β*1 from its latent to its active form [13]. Modifications in the ECM composition could modify such tractions forces and participating in a feedback loop to TGF-*β*1 stimulation on microenvironment cells. GAGs are involved in local concentrations of cytokines and growth factors and reciprocally, TGF-*β*1 enhance GAGs production [114]. Inflammation is responsible for the creation of acidic microenvironment, which may enhance the release of lactates by hematopoietic cells from the clones [115] and activate latent TGF-*β*1 [116], hence further adding to the inflammatory storm in the bone marrow. Another key actor of inflammation and pathogenesis of PMF is neoangiogenesis. VEGF is overproduced in patients [117] and, apart from its role in fibrosis, it plays a pivotal role in the increased vascularization of PMF bone marrow [118]. Taken together, these data strongly suggest that chronic inflammation plays a role in the physiopathology of PMF.

The origin of inflammatory cytokines is mainly represented by pathological clonal cells and remodeling of the microenvironment in a pathological niche clearly involves these clonal cells [6]. Nevertheless, some data raise the question of the role of the inflammatory stimuli in the natural history of PMF. The current concept advocates for a dependence of stromal alterations to cytokines production by the hematopoietic clones. This concept suggests that when clonal disease would be cured, inflammation should stop and will allow an* ad integrum* restitution of the hematopoietic niche. This approach leads to therapy targeting the clonal hematopoietic cells, neglecting other potential target. If evidences are still lacking to attest the nonclonal nature of stromal cells in PMF, some data must be discussed. Cytogenetic-based analyses of bone marrow fibroblasts or MSCs isolated from PMF patients are ancient and based on low-sensitive technics [119–121]. Recently, some data suggested that MSCs from patients could display cytogenetic modification, before culture [122]. Secondly, there is no clear correlation between TGF-*β*1 level and fibrosis: patients without bone marrow fibrosis could exhibit higher level of inflammatory cytokines than patients with marked myelofibrosis [123]. The clinical features of PMF, particularly fibrosis, prominently involve bone marrow but seem to bypass other organs such as the liver or spleen. Could this be due to the presence of activation pathways “exclusive” to bone marrow? The last point questioning this purely reactive conception of bone marrow alterations is the course of fibrosis under therapy. Remissions have been reported in PMF patients after hematopoietic stem cell transplantation [124]. However, its timing is crucial and should be performed before the disease has developed to a very advanced stage. This limitation could explain why the reduction of fibrosis could be significant [124], slow and incomplete [125, 126], or inexistent [127]. Intriguingly, decrease of fibrosis is not correlated with megakaryocytes that are the main source of profibrotic cytokines [126]. Regarding osteosclerosis, data are more homogenous: no improvement is observed under therapy [126, 128, 129]. So, eradicating the hematopoietic clones is not systematically associated with a cure of stromal alterations, keeping open the question of the mutual instructions between hematopoietic and stromal cells.

## 7. Do and How Stroma Alterations Become “Independent” of the Inflammatory Hematopoietic Cell Stimulation?

The concept of MSCs being important effector cells which have the ability to influence the hematopoietic niche has helped to develop new hypothesis and further the current understanding of PMF pathophysiology. Do and how the microenvironment could follow a natural history independently of malignant hematopoietic cells stimulation? Chronic inflammation is typically associated with sustained myeloproliferation and the activation of a number of cellular pathways, which ultimately may trigger DNA damage in hematopoietic cells through ROS accumulation [130]. During inflammation-mediated cells harboring DNA damage may ultimately acquire mutations [131]. Genome wide analysis performed on single MSCs may bring answer to this question. DNA methylation of gene promoters can be promoted by oxidative stress or cytokines like interleukin-6, interleukin-1*β*, or TNF-*α* [132, 133]. Analysis of bone marrow biopsy from PMF patients revealed that hypomethylation of PDGF-*β* gene could be correlated with prognosis and fibrosis [134]. Even if cells harboring methylation modifications cannot be inferred, this data provides evidences of epigenetic modifications occurring during PMF natural history. Inflammation can especially exert its effects on MSCs. Actually, recent results from our lab show that their differentiation abilities could be permanently affected, even in absence of* in vitro* malignant hematopoietic cells stimulation [16]. Mechanisms of these epigenetic modifications are still unclear but may involve inflammation. One form of DNA damage is of particular interest: halogenated cytosine residues. These inflammation damage products have been detected in human leukocytes [135]. The methyl-binding proteins cannot distinguish methylated and halogenated DNA; thus DNMT could be deceived and lead to the accumulation of these analogues within the genome [136]. An initial halogenation, triggered by inflammation, could direct methylation of the complementary DNA strand, resulting in heritable alterations in methylation patterns. In rheumatoid arthritis, synovial fibroblasts exhibit epigenetic alterations thought to be in relation with chronic inflammation, performing an imprinting of their proinflammatory state [137]. Methylation includes not only CpG islands, but also large partially methylated domains and DNA methylation valley (DMV), identified in hematopoietic stem cells [138]. In a mouse model, methylation of this domain can be related to inflammatory exposure resulting in a coordinate aberrant DMV methylation [139]. TGF-*β*1 is a key regulator for DNA methylation through an increase in DNMTs expression and is able to promote methylation in cancer [140, 141]. In renal fibrosis, TGF-*β*1 can induce overproduction of collagen and sclerostin through H3K4 methylation of their promoter [142]. TGF-*β*1-induced profibrotic changes in cell phenotype are accompanied by significant alterations in miR expression profile [143]. In association with TGF-*β*1 challenging, time course of the disease must be taken into account. PMF develops through decades, exposing cellular components to aging, and patient's median age is over 60 years [144]. Analysis of microRNA expression in inflammatory and senescence situation leads to the concept of “inflamm-aging,” involving aberrant expression of microRNA involved in several functions including TGF-*β*1 regulation [145]. MicroRNA expression alteration might occur in MSCs from PMF patients and promote, for instance, modification of TGF-*β*1 expression, osteogenic differentiation, or MSCs trafficking. Altogether, alterations of epigenetic profile of PMF patient's stroma could be promoted by inflammation, resulting in MSC imprinting. With time, inheritance of these modifications could lead to an “autonomous” behavior of MSCs from clonal hematopoietic cells and participate in the disease in a distinctly different manner. Indeed, persistence of a pathologic inflamed stroma, in “absence/decrease” of clonal cells cured by targeted therapies, may explain relapse or drug resistance.

## 8. Conclusion and Perspectives

**Figure 4:**
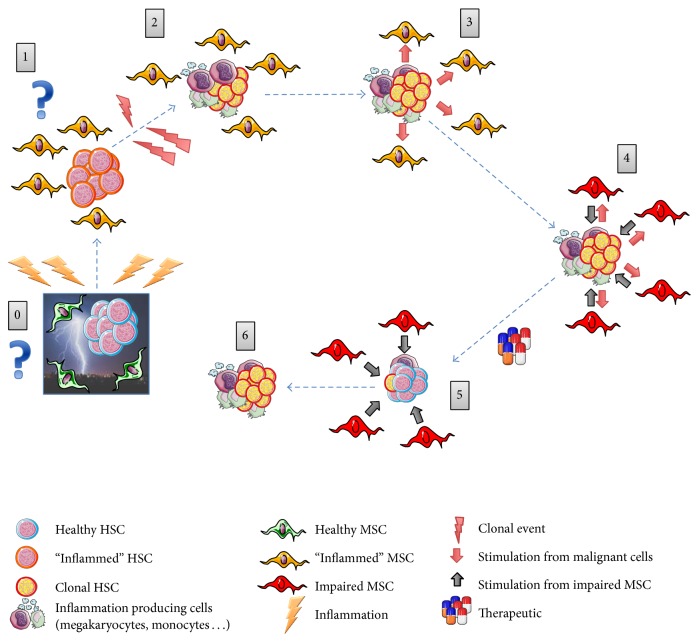
Proposal of a natural history of hematopoietic and stromal cell interactions in bone marrow from PMF patients. The highly inflammed bone marrow environment in PMF is compared to being hit by a “storm” of cytokines [12] (0). (1) This inflammatory environment could involve hematopoietic stem cells (HSCs) and/or mesenchymal stromal cells (MSCs). (2) Clonal events (that would be favored/driven by inflammation (?) [13]) would give rise to clonal hematopoietic cell(s) which will further differentiate into megakaryocytes and monocytes and produce large amount of inflammatory cytokines. (3) These cytokines would modify the bone marrow microenvironment, leading (4) to a permanent impairment of MSCs and a deterioration of the hematopoietic niche. (5) Impaired MSCs would influence malignant hematopoietic cells in an altered crosstalk and over time, MSCs would become inflammatory imprinted. (5) As a result of treatment, the number of malignant hematopoietic cells will reduce. However, the influence of inflammatory imprinted MSCs that would have acquired inherited impaired functions will continue. Unless treated, disease MSCs may continue interacting with HSC and contribute to relapse of the disease (6).

In conclusion, as elegantly proposed by Hasselbalch [64, 131], chronic inflammation may be both an initiator and a driver of clonal evolution in patients with MPNs. In PMF, we suggested that once activated, the stroma is progressively inflammatory-imprinted by clonal hematopoietic cells to an “autonomous” state where it becomes independent of hematopoietic cell stimulation. Therefore, at advanced stage of the disease, this inflammatory vicious circle will become difficult/impossible to break without combined stroma targeted therapies (Figure 4).

The past two decades have provided a wealth of information on how nonresolving inflammation drives a number of widespread chronic diseases including MPNs. Although this knowledge has the potential to open up vast opportunities for new therapeutic advances, the nature of the inflammatory response as a complex system that is critical for normal physiology renders this approach challenging.

Nonetheless, new knowledge about inflammatory signaling, particularly in the setting of MPNs, may provide the promise for new therapeutic options that can successfully meet these challenges. Each of the aspects of pathogenic processes leading to MPNs has unique therapeutic opportunities and challenges. Links between inflammation, JAK2 mutation, and MPNs development have provided a framework for understanding the complex nature of MPNs. However, despite achieving important milestones in the area of MPN research more questions remain unanswered. Important lingering questions include the following: What key triggers lead to activation of inflammation in MPNs? What are the primary danger signals, disease amplifiers, and processes governing sustained chronic inflammation? Can we identify therapeutic targets that are efficacious yet specific enough to avoid unwanted side effects? Does combination therapy where anti-inflammatory drugs are used in combination with JAK inhibitors show greater effectiveness than JAK inhibitors alone, which themselves have anti-inflammatory effects? Given the role for stroma-derived cytokines in protecting the malignant clones against JAK2-directed therapy [146], how could the stromal niche be manipulated to target the clone and to restore normal hematopoiesis? Answering these questions should increase our understanding about the pathogenesis of MPNs and should provide exciting targets and new treatment options.

Another important question concerns the timing of when to begin the treatment of patients? To be efficient, inflammatory/antifibrotic strategies must not only limit the progression of inflammation/fibrosis by eliminating the source of promoting agents but also counteract damaged BM stroma by promoting repair processes. Similarly to stem cell transplantation, such treatments must be as early as possible, before the disease has developed to a very advanced stage, to avoid the “irreversible” inflammatory imprinting of the stroma and to be given in combination with drugs aiming at prohibiting the hematopoietic clone. In addition to treatments such as JAK inhibitors and anti-inflammatory agents (including immunomodulatory agents such as Interferon-alpha), as monotherapy or in combination, epigenetic modifiers have also been proposed. From a mechanistic viewpoint, it seems plausible that epigenetic therapy directed against DNA methylation, histone acetylation, and microribonucleic acids (microRNAs) might indeed improve clinical outcomes and alleviate PMF-related symptoms [149]. Recently, Tibes and Mesa suggested the concept of targeting Sonic hedgehog (Shh) signalling in PMF since inhibitors of this pathway (sonidegib) have shown preliminary activity (including reduction of fibrosis) as single agents or in combination with ruxolitinib in preclinical and clinical studies [150]. It has been recently reported that Shh signalling from bone marrow-derived mesenchymal stromal cells of MDS patients plays a role in the survival advantage of myelodysplastic cells by modulating DNA methylation [151]. Taking into account the role of the Sonic Hedgehog signalling in modifying the expression of genes modulated in PMF MSCs (our data), targeting Shh in stromal cells could be a promising approach to reduce inflammation in PMF and, in association (or not?) with JAK2 inhibitors, to better control the hematopoietic clonal proliferation.
